# Supplementary material for: The microbiome biomarkers of pregnant women’s vaginal area predict preterm prelabor rupture in Western China
Source: Front Cell Infect Microbiol. 2024 Oct 31;14:1471027. doi: 10.3389/fcimb.2024.1471027 (PMC11560878; doi:10.3389/fcimb.2024.1471027)
Supplement: Supplementary file 1 [file DataSheet1.zip › compare_1/Community/Pieplot/family/sample/P32.family.pieplot.pdf]

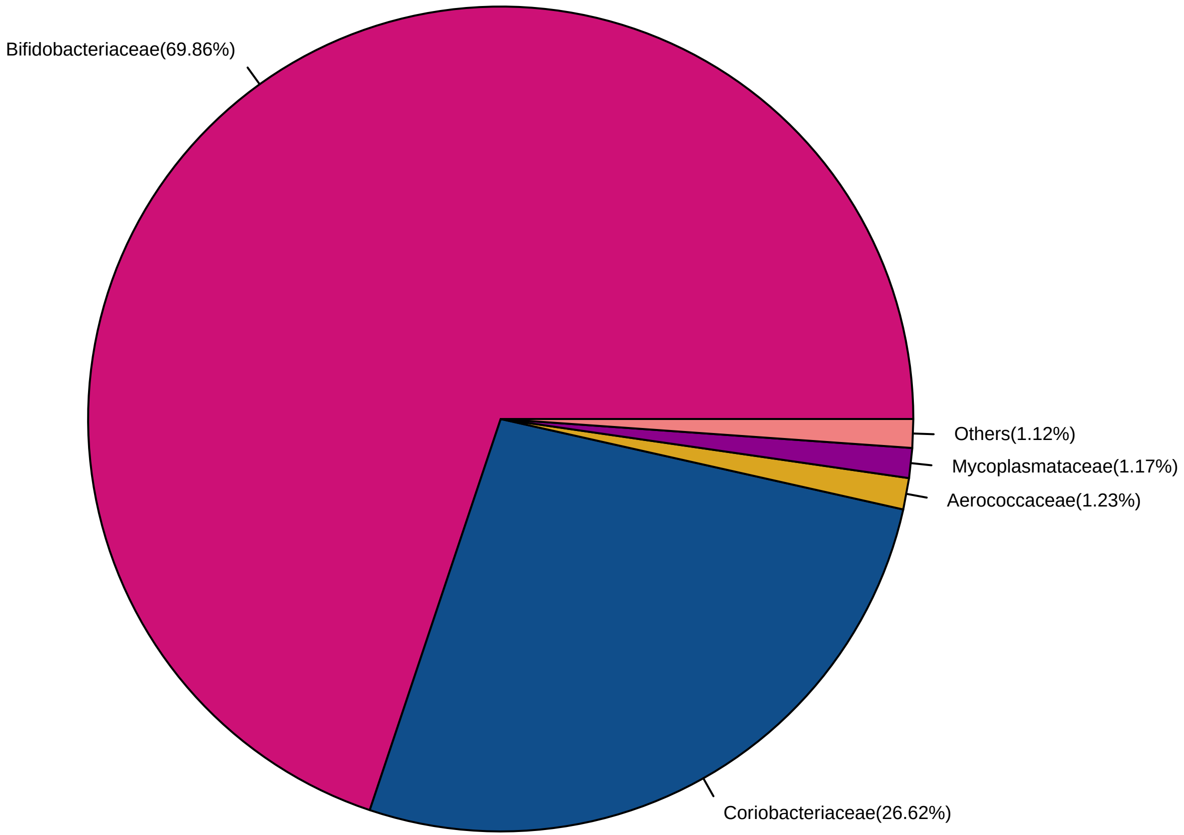

Bifidobacteriaceae(69.86%)
Others(1.12%)
Mycoplasmataceae(1.17%)
Aerococcaceae(1.23%)
Coriobacteriaceae(26.62%)
